# Utility of Endoscopic Examination in the Diagnosis of Acute Graft-versus-Host Disease in the Lower Gastrointestinal Tract

**DOI:** 10.1155/2017/2145986

**Published:** 2017-10-02

**Authors:** Kosuke Nomura, Toshiro Iizuka, Daisuke Kaji, Hisashi Yamamoto, Yasutaka Kuribayashi, Masami Tanaka, Tsukasa Furuhata, Satoshi Yamashita, Daisuke Kikuchi, Akira Matsui, Toshifumi Mitani, Yasunori Ota, Shuichi Taniguchi, Shu Hoteya

**Affiliations:** ^1^Department of Gastroenterology, Toranomon Hospital, Tokyo, Japan; ^2^Department of Hematology, Toranomon Hospital, Tokyo, Japan; ^3^Department of Pathology, Research Hospital, The Institute of Medical Science, The University of Tokyo, Tokyo, Japan

## Abstract

**Background and Aims:**

We retrospectively investigated the incidence of acute graft-versus-host disease (GVHD) in the lower gastrointestinal (GI) tract and the diagnostic accuracy of endoscopy.

**Methods:**

Of 1231 patients who underwent allogeneic hematopoietic stem cell transplantation between January 2005 and December 2014, 186 of whom underwent colonoscopy and biopsy and had no cytomegalovirus infection. The endoscopic findings and histologic diagnosis from these 186 patients were retrospectively analyzed.

**Results:**

Based on the histopathological findings, 171 patients were diagnosed with GVHD, accounting for 13.9% of all transplant recipients. Useful endoscopic findings for the diagnosis of GVHD were atrophy of the ileocecal valve and villous atrophy in the terminal ileum and tortoise shell-like mucosae, edema, and low vascular permeability in the colon. Even when no mucosal abnormality was observed, the incidence of GVHD was 78.9% in the terminal ileum and 75.0% in the colon. Furthermore, patients with mucosal exfoliation, although infrequent, were all diagnosed with grade 3/4 GVHD.

**Conclusions:**

It is important to perform endoscopy proactively for the early diagnosis of GVHD, and biopsy should be performed even when no abnormality is observed. In addition, because patients with mucosal exfoliation are extremely likely to have grade 3/4 GVHD, early treatment should be initiated.

## 1. Introduction

Early diagnosis and treatment are essential for graft-versus-host disease (GVHD), which is a serious complication that influences the prognosis of patients after allogeneic transplantation [1]. However, because GVHD is frequently accompanied by various infections that developed after hematopoietic stem cell transplantation as well as by disorders induced by nonimmunological mechanisms, it is difficult to identify GVHD based solely on clinical symptoms [2]. Therefore, when making a diagnosis of GVHD in the gastrointestinal (GI) tract, it is important to perform endoscopy and diagnostic histopathological examination of biopsy specimens [3], but only a few studies have reported the endoscopic feature of GVHD in detail.

In this study, we therefore retrospectively investigated the incidence and endoscopic features of GI GVHD that had occurred within 100 days of hematopoietic stem cell transplantation and analyzed the association between the endoscopic features and histopathological GVHD.

## 2. Patients and Methods

### 2.1. Patients

**Figure 1:**
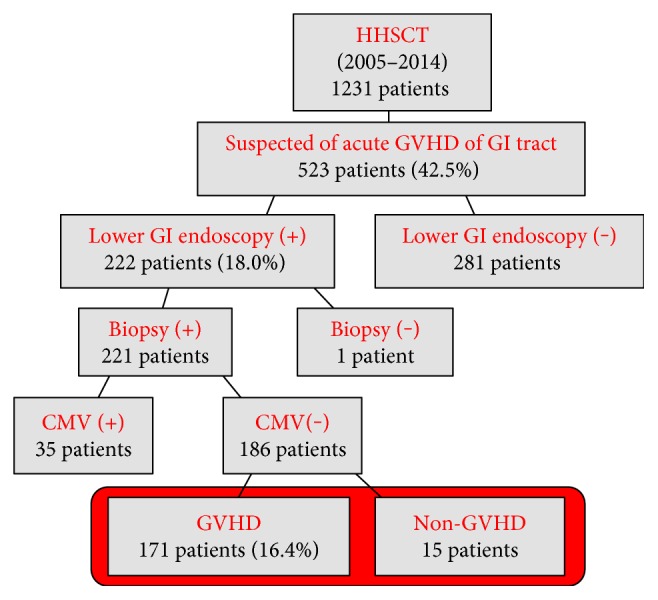
Study flow diagram. HHSCT: homologous hematopoietic stem cell transplantation; GI: gastrointestinal; CMV: cytomegalovirus.

Of 1231 patients who underwent allogeneic hematopoietic stem cell transplantation between January 2005 and December 2014, 523 developed GI symptoms indicative of GVHD such as diarrhea and poor appetite and 222 subsequently underwent colonoscopy. After excluding 35 patients with cytomegalovirus (CMV) infection, 186 patients who had also undergone biopsy for histopathological assessment were enrolled in this study (Figure 1). Some patients underwent identical endoscopic examinations more than twice within 100 days of transplantation. In those patients, the results from the first examination were analyzed in this study.

### 2.2. Methods

**Figure 2:**
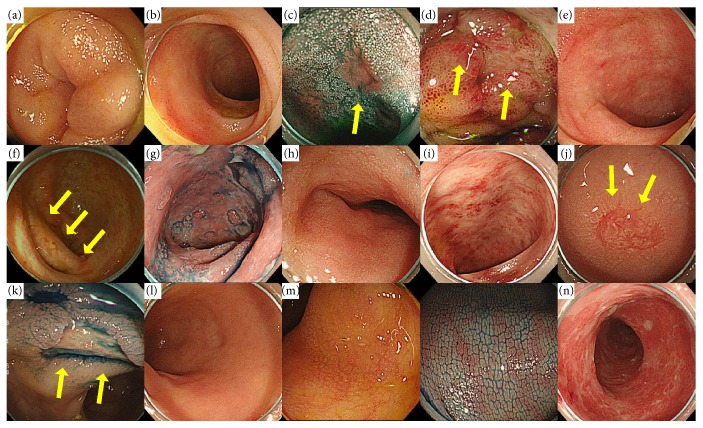
Examples for endoscopic findings of lower GI: (a) edema, (b) redness, (c) erosion, (d) ulcer, (e) villous atrophy, (f) atrophy of the ileocecal valve, and (g) mucosal exfoliation in the terminal ileum and (h) edema, (i) redness, (j) erosion, (k) ulcer, (l) low vascular permeability, (m) tortoise shell-like mucosae, and (n) mucosal exfoliation in the colon.

In each case, various endoscopic findings (edema, redness, erosion, ulcer, villous atrophy, atrophy of the ileocecal valve, and mucosal exfoliation in the terminal ileum and edema, redness, erosion, ulcer, low vascular permeability, tortoise shell-like mucosae, and mucosal exfoliation in the colon) were extracted retrospectively (Figure 2), and their association with histopathological GVHD was investigated by calculating sensitivity, specificity, and positive predictive values. A similar analysis of grade 3/4 GVHD was performed. Histopathological diagnosis of GVHD was performed according to the grading criteria developed by Sale et al. [4] (Table 1). In addition, when patients underwent multiple biopsies, the highest histopathological grade was used in this study. It should be noted here that biopsy was performed only when the number of platelets was ≥50,000 cells/mm^3^.

## 3. Results

### 3.1. Incidence

Based on histopathological findings in the lower GI tract, GVHD was diagnosed in 171 patients, accounting for 91.9% of the patients who had undergone endoscopy and 16.4% of all transplant recipients. Sixty patients had GVHD only in the lower GI tract and not in other organs.

### 3.2. Patient Background

Endoscopy was performed in 122 men and 64 women (average age, 50.6 years; range, 19–82 years) within approximately 41.3 (13–100) days after transplantation. The primary disease was acute leukemia in 120 patients, malignant lymphoma in 38, myelodysplastic syndrome in 16, chronic myelogenous leukemia in 5, and other in 7. The source of stem cells for transplantation was umbilical cord blood in 125 patients, bone marrow in 36, and peripheral blood from a relative in 25. All patients underwent additional treatment to prevent GVHD, such as combination therapy with tacrolimus (FK506) and mycophenolate mofetil (MMF) in 72 patients; combination therapy with methotrexate (MTX), MMF, and FK506 or cyclosporin A (CyA) in 57; and monotherapy with CyA in 57. Biopsies were conducted at 4.9 sites on average (Table 2). In addition, a colonoscope was inserted up to the terminal ileum in 154 (82.8%) patients, to the right-side colon in 17 (9.1%), and to the left-side colon in 15 (8.0%).

### 3.3. Histopathological Staging of GVHD

Histopathological severity of GVHD was grade 1 in 52 (28.0%) patients, grade 2 in 36 (19.4%), grade 3 in 45 (24.2%), and grade 4 in 38 (20.4%).

### 3.4. Positive Biopsy Rate

The rate of positive biopsy by a GI region was 90.4% for the terminal ileum and 94.4% for the colon (Table 3). More specifically, the left- and right-side colon and terminal ileum all had similar rates (around 90%), with no significant difference between the anatomical regions. Reanalysis of actual GVHD cases revealed that 97.3% of the patients were diagnosed with GVHD based on colonoscopy of the left side of the colon alone, whereas the remaining 2.7% were diagnosed based on colonoscopy up to the right side of the colon or terminal ileum, and GVHD was negative in the left side of the colon. In the analysis of anatomical sites with the highest grade in each patient, the most severe form of GHVD was found frequently in the following order: left side of the colon < right side of the colon < terminal ileum. This shows that the severity of GVHD tended to increase as it gets deeper.

### 3.5. Terminal Ileum

GVHD was present in the terminal ileum of 122 patients, one of whom was diagnosed based on the assessment of the terminal ileum alone. Frequent endoscopic findings were atrophy of the ileocecal valve (68.0%) and villous atrophy (49.6%). No abnormal findings were observed in 30 (22.2%) patients. Sensitivity toward GVHD was low for every endoscopic feature, but specificity and positive predictive value were 100% for villous atrophy, ulcer, and mucosal exfoliation and ≥80% for edema, redness, and erosion. In addition, GVHD was diagnosed in 80% of the patients with no abnormal findings. Reanalysis of grade 3/4 GVHD cases showed a high positive predictive value of approximately 90% for mucosal exfoliation (Table 4).

### 3.6. Colon

GVHD was detected in the colon of 170 patients. Frequent endoscopic findings were low vascular permeability (80.0%), edema (73.3%), and tortoise shell-like mucosae (67.6%). Specificity was 100% for ulcer and mucosal exfoliation and 93.3% for erosion, and positive predictive value was ≥90% for all endoscopic findings, showing high diagnostic performance. GVHD was diagnosed in 75% of 12 patients with no abnormal findings. Analysis of grade 3/4 GVHD cases revealed a high positive predictive value of 93.3% for mucosal exfoliation and 81.3% for ulcer (Table 4).

### 3.7. Treatment Efficacy

Treatment efficacy was observed in 75.4% of all patients. Potent therapy with, for example, antithymocyte globulin (ATG) or infliximab tended to be indicated for histopathologically severe GVHD, showing a correlation with actual clinical practice. A comparison by a disease stage showed that treatment efficacy was as high as 92.3% in grade 1 GVHD but decreased gradually to 86.1% in grade 2, 80.0% in grade 3, and markedly to 36.8% in grade 4 (Table 5). Comparative analyses with endoscopic findings revealed that treatment efficacy was clearly low at 50.0% in 26 patients with mucosal exfoliation.

## 4. Discussion

GVHD is a disease caused by donor lymphocytes recognizing host histocompatibility antigens as foreign and attacking them immunologically. In definition, GVHD occurs within 100 days of transplantation [5]. Primary target organs are the skin, liver, and GI tract, and characteristic clinical symptoms include maculopapular rash, nausea, vomiting, diarrhea, and jaundice. The pathogenesis of GI GVHD is thought to be direct mucosal damage in the GI tract made directly by donor cytotoxic T cells and tissue damage made by cytokines secreted by the T cells [1].

The incidence of acute GI GVHD is estimated to be 30–60% of all transplant recipients [6]. In general, various GI symptoms such as watery diarrhea, nausea, vomiting, and poor appetite start 1-2 weeks after the appearance of skin lesions. Abdominal pain and melena also develop as the disease progresses, and paralytic ileus is observed in severe GVHD cases. In more severe cases, sepsis may trigger endotoxin shock, potentially causing death. Even though GVHD is a life-threatening complication, diagnostic specificity based solely on clinical symptoms was reported to be 50% or even below in patients with GVHD [7].

Biopsy is essential in making a diagnosis of GVHD, and specimens are taken from organs such as the skin, liver, and GI tract. The rate of positive biopsy was 46% for the skin but was as high as 58% for the upper and lower GI tract, indicating the validity of GI endoscopy in diagnostic biopsy [6]. In this study, histopathological diagnosis of lower GI GVHD was made in 91.9% of the patients suspected of having GVHD. At our hospital, endoscopic examination through the GI tract is performed for the definitive diagnosis of GVHD as soon as the disease is suspected, which apparently contributed to the high diagnostic rate in this study.

GVHD is histopathologically characterized by the apoptosis of crypt epithelial cells accompanied by the infiltration of lymphocytes [8]. The epithelial cells in the crypt base region, from where the intestinal epithelium proliferates, appear to be the target of GVHD [9]. The clinical course of GVHD involves the development of crypt abscess due to neutrophil infiltration, crypt loss, and eventually mucosal exfoliation. Apoptosis of crypt epithelial cells is observed occasionally in the biopsy specimens of the mucosa that appear normal, providing useful information for the early diagnosis of GVHD [4].

The differential diagnosis includes regimen-related toxicity, CMV and other viral infections, TMA (thrombotic microangiopathy), bacterial enteritis such as pseudomembranous enterocolitis, and *Candida* and other fungal enteritis [10]. Before making a diagnosis of GVHD, it is important to remember that CMV infection and the effect of pretransplantation chemotherapy or radiotherapy, which lasts for about 20 days after transplantation, can induce apoptosis [11]. After allogeneic transplantation, agranulocytosis triggered by pretreatments lasts for 2-3 weeks, and cellular immunodeficiency induced by immunosuppressants continues even after engraftment, increasing the risk of infection and the resulting mortality rate. These complications are closely associated with the recurrence risk of leukemia, often making their management difficult. While the risk of mortality from GVHD increases when immunosuppressants are not potent enough, superpotent immunosuppressants increase the risks of recurrent leukemia and infection. It is essential to balance the risks of GVHD and infections to improve treatment efficacy after allogeneic transplantation.

In this study, 16.4% of the 1231 transplant recipients developed GVHD in the lower GI tract. The known common sites of lower GI GVHD are the terminal ileus, cecum, and ascending colon [12]. However, no significant difference was observed between the left- and right-side colon and the terminal ileum in this study (Table 3).

The known characteristic endoscopic features of GVHD in the terminal ileum are villous atrophy, redness, edema, erosion, and shallow and wide ulcer-like lesions [12], but these features vary extensively and lack specificity. In this study, atrophy of the ileocecal valve and villous atrophy were frequent endoscopic findings with high specificity and positive predictive value. In particular, atrophy of the ileocecal valve has not been reported previously, and the high incidence rate (approximately 70%) and positive predictive value (approximately 90%) in this study suggest that atrophy of the ileocecal valve can be a useful diagnostic marker for lower GI GVHD.

The known characteristic endoscopic findings of GVHD in the colon are orange peel appearance and tortoise shell-like mucosae [13], the latter of which is a regularly mesh pattern appearing in the mucosa of the colon and can be visualized clearly by indigo carmine dye spraying. It represents highly advanced diffuse edematous changes in the mucosa and is histologically associated with edema in the lamina propria. The enlargement of the lamina propria due to edema makes cryptic density sparse in the colon and emphasizes innominate grooves and the opening of crypts, leading to the appearance of the tortoise shell-like mucosae. In this study, the incidence and positive predictive value of the tortoise shell-like mucosae were both high, suggesting that it is a characteristic endoscopic finding of lower GI GVHD. The incidence rate and positive predictive value were also high for low vascular permeability and mucosal edema.

Also in this study, 26 patients had mucosal exfoliation (18 in the terminal ileum and 15 in the colon). Despite the relatively low incidence rate, the positive predictive value of mucosal exfoliation was as high as 92.3% for grade 3/4 GVHD, indicating that this endoscopic feature is an extremely important predictor of severe GVHD. Indeed, only about 50% of the patients with mucosal exfoliation responded to treatment in this study. Therefore, when endoscopic findings include mucosal exfoliation, early intervention should be initiated even before obtaining biopsy results.

In addition, attention must be paid to patients with no abnormal endoscopic findings because in a previous study, GVHD was histopathologically diagnosed in the areas appearing virtually normal in endoscopy [2]. Also in this study, GVHD was histopathologically diagnosed in 78.9% of the terminal ileum and 75.0% of the colon where no abnormal endoscopic features were observed. Therefore, even when patients present with seemingly normal gastrointestinal mucosa, biopsy should be performed whenever possible.

In this study, we also investigated whether the deep insertion of a colonoscope is necessary. In clinical practice, it is often difficult to insert a colonoscope all the way to the terminal ileum in transplant recipients because of poor general conditions and insufficient bowel preparation. In this study, lower GI GVHD was diagnosed at a rate of 91.2% based solely on biopsy assessment of the left-side colon, suggesting that colonoscopy should be performed regardless of patient performance status. However, because the terminal ileum is often most severely affected by GVHD, colonoscopy should include the terminal ileum if possible.

This study has several limitations. First, this is a retrospective single-institution study with a limited number of patients. Secondly, the interpretation of endoscopic findings was performed by one examiner. Thirdly, GI endoscopy was not performed in all transplant recipients, generating sampling bias. Therefore, we plan to perform a prospective study with a large number of patients in the future.

In summary, lower GI GVHD was diagnosed histopathologically in 91.9% of the patients who had undergone endoscopy and 13.9% of all transplant recipients. Useful endoscopic findings for the diagnosis of GVHD were atrophy of the ileocecal valve and villous atrophy in the terminal ileum and tortoise shell-like mucosae, edema, and low vascular permeability in the colon. In addition, although rare, mucosal exfoliation was highly correlated with grade 3/4 GVHD, necessitating the initiation of early intervention even before obtaining biopsy results.

## Figures and Tables

**Table 1:** Definition and the histological grading system for acute GI GVHD.

Grade	Histological feature
I	Single-cell necrosis (apoptosis) noted on medium power
II	Evidence of epithelial damage by crypt/grandular abscesses, epithelial flattening, or glandular/crypt dilation
III	Dropout of one or more crypts/grands
IV	Total epithelial denudation

**Table 2:** Patient characteristics.

Number	186
Average age, year (range)	50.6 (19–82)
Gender	
Male	122
Female	64
Average inspection days after transplantation	
CS	41.3 (13–100)
Average number of biopsy specimens	4.9
Primary disease	
Acute leukemia	120
Malignant lymphoma	38
Myelodysplastic syndrome	16
Chronic myelogenous leukemia	5
Others	7
Stem cell source	
Cord blood stem cell transplantation	125
Bone marrow transplantation	36
Alloperipheral blood stem cell transplantation	25
Previous treatment	
FLU-based regimen	161
CY/TBI or CY/BU	25
Preservation of GVHD	
FK + MMF	72
MTX + CyA/FK/MMF	57
CyA or FK	57
Extent of endoscopic insertion	
Up to the left-side colon	15
Until the right-side colon	17
Terminal ileum	154

FLU: fludarabine; CY: cyclophosphamide; TBI: total body irradiation; BU: busulfan; CyA: cyclosporin A; MTX: methotrexate; MMF: mycophenolate mofetil.

**Table 3:** The rate of positive biopsy.

*Terminal ileum*	90.4%
*Colon*	94.4%
Right-side colon	91.5%
Left-side colon	91.7%
Rectum only	87.7%

**Table 4:** Summary of outcomes of lower GI.

	Accuracy (%)	Sensitivity (%)	Specificity (%)	PPV (%)	PPV (%) (Grade III or IV)
Terminal ileum					
Edema	36.3	40.0	81.8	96.2	65.4
Redness	40.0	44.0	81.8	96.5	66.7
Erosion	28.9	31.2	90.9	97.5	70.0
Ulcer	5.2	5.6	100	100	71.4
Villous atrophy	49.6	54.4	100	100	67.6
Atrophy of the ileocecal valve	69.2	71.3	55.6	87.7	50.6
Mucosal exfoliation	12.3	14.4	100	100	88.9
No remarkable findings	22.2	24.0	27.3	78.9	7.9
Colon					
Edema	73.0	75.4	53.3	94.9	50.7
Redness	52.2	53.2	60.0	93.8	59.8
Erosion	38.2	40.9	93.3	98.6	74.6
Ulcer	8.6	9.4	100	100	81.3
Low vascular permeability	80.0	81.3	33.3	93.3	50.3
Tortoise shell-like mucosae	67.6	49.7	53.3	94.4	48.4
Mucosal exfoliation	8.1	8.8	100	100	93.3
No remarkable findings	6.5	7.1	80.0	75.0	0.0

PPV: positive predictive value.

**Table 5:** Histological grade and effectiveness of treatment in patients with acute GI GVHD.

GVHD grade	No treatment or BDP	+FK, PSL, and mPSL	+ATG and infliximab	Total
Grade I	100% (19/19)	87.0% (20/23)	90.0% (9/10)	92.3% (48/52)
Grade II	100% (9/9)	88.2% (15/17)	70.0% (7/10)	86.1% (31/36)
Grade III	87.5% (7/8)	90.5% (19/21)	62.5% (10/16)	80.0% (36/45)
Grade IV	100.0% (3/3)	44.4% (8/18)	17.6% (3/17)	36.8% (14/38)
Total	97.4% (38/39)	78.5% (62/79)	54.7% (29/53)	75.4% (129/171)

BDP: beclometasone dipropionate; PSL: prednisolone; mPSL: methylprednisolone; ATG: antithymocyte globulin.
